# Energy Redistribution Following CO_2_ Formation on Cold Amorphous Solid Water

**DOI:** 10.3389/fchem.2021.827085

**Published:** 2022-02-08

**Authors:** Meenu Upadhyay, Markus Meuwly

**Affiliations:** Department of Chemistry, University of Basel, Basel, Switzerland

**Keywords:** reactive molecular dynamics, amorphous solid water, interstellar chemistry, energy redistribution, CO_2_ formation

## Abstract

The formation of molecules in and on amorphous solid water (ASW) as it occurs in interstellar space releases appreciable amounts of energy that need to be dissipated to the environment. Here, energy transfer between CO_2_ formed within and on the surface of amorphous solid water (ASW) and the surrounding water is studied. Following CO(^1^Σ^+^) + O(^1^D) recombination the average translational and internal energy of the water molecules increases on the ∼10 ps time scale by 15–25% depending on whether the reaction takes place on the surface or in an internal cavity of ASW. Due to tight coupling between CO_2_ and the surrounding water molecules the internal energy exhibits a peak at early times which is present for recombination on the surface but absent for the process inside ASW. Energy transfer to the water molecules is characterized by a rapid ∼10 ps and a considerably slower ∼1 ns component. Within 50 ps a mostly uniform temperature increase of the ASW across the entire surface is found. The results suggest that energy transfer between a molecule formed on and within ASW is efficient and helps to stabilize the reaction products generated.

## 1 Introduction

The motion of adsorbates in and on amorphous solid water (ASW) is essential for chemistry at astrophysical conditions. Typically, bulk water is present in the form of ASW which is the main component of interstellar ices. (Hagen et al., 1981). The structure of ASW is usually probed by spectroscopic measurements (Hagen et al., 1981; Jenniskens and Blake, 1994) although interference-based methods have also been employed. (Bossa et al., 2012). ASWs are porous structures characterized by surface roughness and internal cavities of different sizes which can retain molecular or atomic guests. (Bar-Nun et al., 1987). Under laboratory conditions the water ices have been reported to be porous (He et al., 2016; Kouchi et al., 2020) or non-porous (Oba et al., 2009; He et al., 2016; Kouchi et al., 2020) ASW whereas the morphology of ices in the interstellar medium is more debated. (Keane et al., 2001; Kouchi et al., 2021).

The high porosity of ASW (Bossa et al., 2014; Bossa et al., 2015; Cazaux et al., 2015) makes it a good catalyst for gas-surface reactions involving oxygen (Ioppolo et al., 2011a; Romanzin et al., 2011; Chaabouni et al., 2012; Minissale et al., 2013a; Dulieu et al., 2017; Pezzella et al., 2018; Pezzella and Meuwly, 2019; Christianson and Garrod, 2021), hydrogen (Hama and Watanabe, 2013), carbonaceous (Minissale et al., 2013b; Minissale et al., 2016a; Qasim et al., 2020; Molpeceres et al., 2021) and nitrogen-containing (Minissale et al., 2014) species and helps maintaining those species on (Minissale et al., 2019) or inside ASW. (Minissale et al., 2016b; Tsuge et al., 2020). This increases the probability for the reaction partners to diffuse to locations for collisions and association reactions to occur. As the diffusivity of individual atoms and small molecules has been established from both, experiments and simulations, (Minissale et al., 2013b; Lee and Meuwly, 2014; Pezzella et al., 2018), this is a likely scenario for formation of molecules on and within ASW.

Earlier thermoluminescence experiments suggested that the O(^3^P)+CO(^1^Σ^+^) reaction with both reaction partners in their electronic ground state yields excited ${CO}_{2}^{*}$ which, after emission of a photon, leads to formation of CO_2_. (Fournier et al., 1979). Such a process has also been proposed to occur on interstellar grains (Ruffle and Herbst, 2001) and has been confirmed experimentally (Minissale et al., 2013b) with an estimated entrance barrier of 0.014–0.103 eV for the process on ASW, compared with a value of 0.3 eV from high-level electronic structure calculations. (Veliz et al., 2021). The surrounding water matrix provides the necessary coupling (Roser et al., 2001) to facilitate relaxation of the ^3^A’ or ^3^A^*″*^ states of CO_2_ to the ^1^A’ ground state (correlating with linear Σg+1 ). The presence of an entrance barrier for the O(^3^P)+CO(^1^Σ^+^) reaction has one led to consider the alternative CO + OH pathway for CO_2_ formation. (Watanabe and Kouchi, 2002; Ioppolo et al., 2011b). This was, however, reconsidered to yield the HOCO intermediate in such environments in more recent experiments. (Qasim et al., 2019). Also, the reaction products of the CO + OH reaction have been found to depend on the experimental conditions. (Oba et al., 2010; Noble et al., 2011). As the CO + OH reaction also appears to have a barrier in the entrance channel, (Noble et al., 2011), attention has recently shifted to the HOCO + H reaction for CO_2_ formation. (Qasim et al., 2019).

For adsorbed species to react on ASW they need to be able to diffuse. This has been demonstrated from MD simulations with diffusion coefficients and desorption energies consistent with experiments. (Lee and Meuwly, 2014; Ghesquière et al., 2015). Atomic oxygen (Pezzella et al., 2018) on ASW experiences diffusional barriers between *E*_dif_ = 0.2 kcal/mol and 2 kcal/mol (100–1000 K) compared with values of $E_{\text{dif}}=990_{-360}^{+530}$ K determined from experiments. (Minissale et al., 2016b). For CO, MD simulations reported (Pezzella and Meuwly, 2019) desorption energies between 3.1 and 4.0 kcal/mol (1560–2012 K or 130–170 meV), compared with 120 meV from experiments. (Karssemeijer et al., 2013). It was also found that the CO desorption energy from ASW depends on CO coverage with ranges from *E*_des_ = 1700 K for low to 1000 K for high coverage (He et al., 2016) which is consistent with the simulations. (Pezzella and Meuwly, 2019). On non-porous and crystalline water surfaces submonolayer desorption energies for CO are 1307 and 1330 K ( ∼115 meV), respectively. (Noble et al., 2012). Experimental diffusional barriers range from 350 ± 50 K (Kouchi et al., 2020) to 490 ± 12 K. (He et al., 2018).

As such association reactions are in general exothermic, the energy released needs to be transferred to environmental degrees of freedom for the reaction products to stabilize. This is the quest of the present work which investigates the time scale and degrees of freedom to receive the energy liberated for the O(^1^D)+CO(^1^Σ^+^) reaction to form ground state CO_2_( Σg+1 ). The chemical precursors for formation of CO_2_ are believed to be carbon monoxide and atomic oxygen and the CO + O reaction has been proposed as a non-energetic pathway, close to conditions in interstellar environments, for CO_2_ formation 20 years ago from experiments involving a water-ice cap on top of CO and O deposited on a copper surface. (Roser et al., 2001). Formation of CO_2_( Σg+1 ) from ground state CO(^1^Σ^+^) and electronically excited O(^1^D) is barrierless. The excited atomic oxygen species can, for example, be generated from photolysis of H_2_O (Stief et al., 1975) which has a radiative lifetime of 110 min (Garstang, 1951). An alternative pathway proceeds via electron-induced neutral dissociation of water into H_2_ + O(^1^D). (Schmidt et al., 2019). In the presence of CO formation of CO_2_ in cryogenic CO/H_2_O films was observed. (Schmidt et al., 2019).

After recombination O(^1^D)+CO(^1^Σ^+^) → CO_2_( Σg+1 ) the product is in a highly vibrationally excited state. For it to stabilize, excess internal energy needs to be channeled into the environment which is the ASW. The present work characterizes and quantifies energy relaxation of the CO_2_( Σg+1 ) product into internal and translational degrees of freedom of the surrounding water matrix. First, the methods used are described. Then, results are presented and discussed. Finally, conclusions are drawn.

## 2 Computational Methods

**FIGURE 1:**
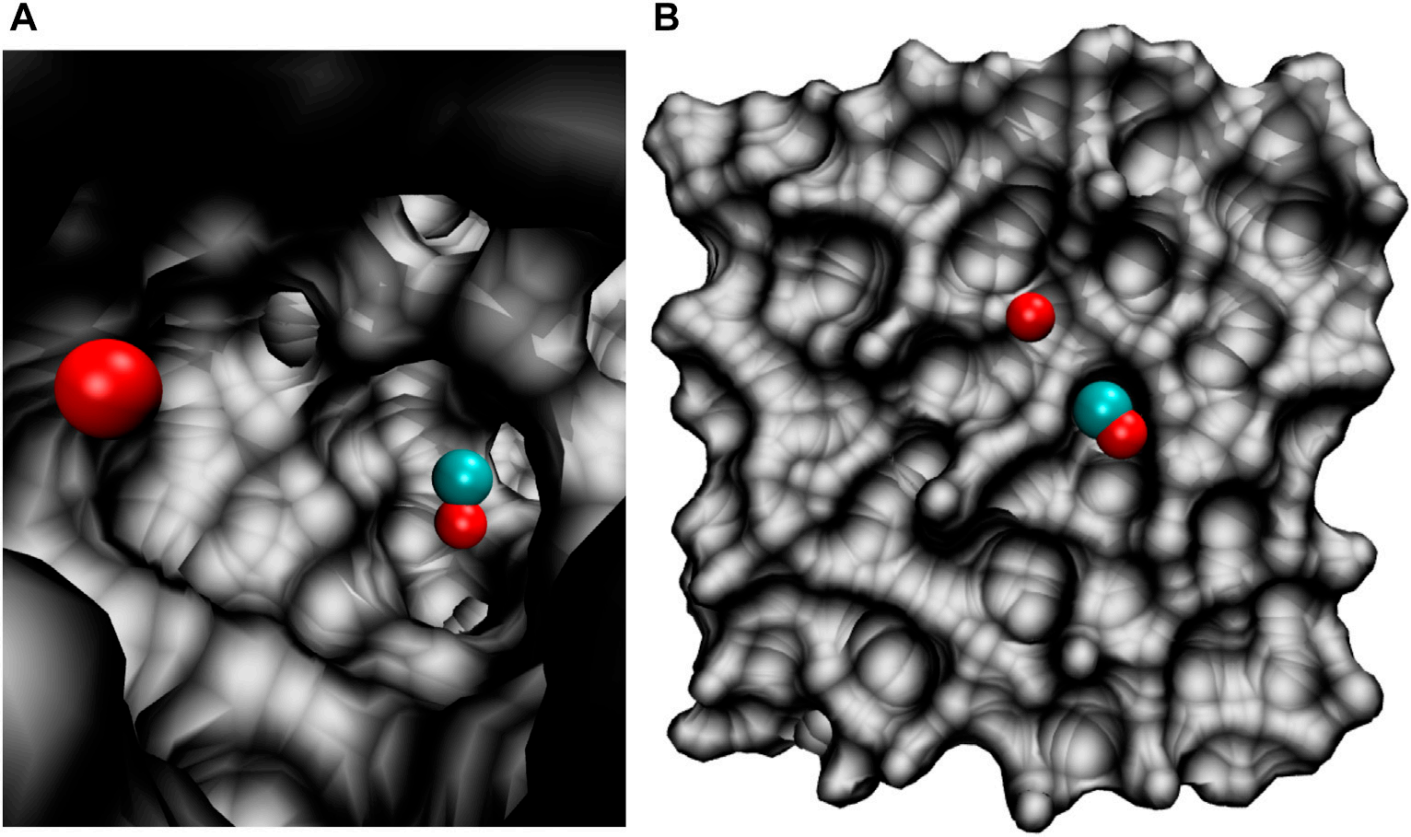
The simulation system for studying the O(^1^D)+CO(^1^Σ^+^)→ CO_2_( Σg+1 ) recombination reaction. **(A)**: CO and O trapped inside a cavity of ASW; **(B)**: CO and O on the top of the ASW surface.

All molecular dynamics (MD) simulations were carried out using the CHARMM suite of programs (Brooks et al., 2009) with provisions for bond forming reactions through multi state adiabatic reactive MD (MS-ARMD). (Nagy and Yosa Reyes, 2014). The simulation system, Figure 1, consisted of an equilibrated cubic box of amorphous solid water with dimension 31 × 31 × 31 Å^3^ containing 1000 water molecules. As all bonds and angles are flexible, the simulations were run with a time step of Δ*t* = 0.1 fs and the non-bonded cutoff was at 13 Å. Simulations were started from an existing, equilibrated ASW structure (Pezzella et al., 2018; Pezzella and Meuwly, 2019; Upadhyay et al., 2021) by adding CO_A_ and O_B_ inside (Figure 1A) or on top of (Figure 1B) ASW.

In the following, the coordinates are the CO stretch *r*, the separation *R* between the center of mass of CO_A_ and O_B_ and *θ* is the O_A_CO_B_ angle. In addition, the C–O_B_ separation will be considered where appropriate. Initial conditions were generated for a grid of angles *θ* and separations *R* and simulations were carried out to obtain initial coordinates and velocities for each of the grid points. With constrained CO and O position, first 750 steps of steepest descent and 100 steps Adopted Basis Newton-Raphson minimization were carried out, followed by 50 ps heating dynamics to 50 K. Then, 100 ps equilibration dynamics was carried out. From each of the runs coordinates and velocities were saved regularly to obtain initial conditions for each combination of angle and distance. Production simulations 500 ps or 6 ns in length were then run from saved coordinates and velocities in the *NVE* ensemble. Data (energies, coordinates and velocities) were saved every 1000 steps for subsequent analysis.

Water was described by a reparametrized, (Burnham et al., 1997; Plattner and Meuwly, 2008), flexible KKY (Kumagai, Kawamura, Yokokawa) model. (Kumagai et al., 1994). The typical water modes that couple in the $∼2000\;{cm}^{-1}$ region relevant in the present work are the water bend (1,600 cm^−1^) and the framework rotation (600 cm^−1^) as was also found for the vibrational relaxation of cyanide in water. (Lee and Meuwly, 2011). To describe CO_A_ + O_B_ recombination to form CO_2_ the Morse-Morse-Harmonic (MMH) parametrization was employed. (Upadhyay et al., 2021). This model treats the two CO bonds with a Morse potential and the OCO bend as a harmonic function. MMH is a computationally efficient model (fitted to MRCI/aug-cc-pVTZ data), which yields results for recombination probabilities on ASW comparable to a more elaborate reproducing kernel Hilbert space (RKHS) representation with an exothermicity of –7.27 eV (Veliz et al., 2021; Upadhyay et al., 2021).

For CO_2_, the partial charges were *q*_O_ = − 0.3*e* and *q*_C_ = 0.6*e* with standard van der Waals parameters from CHARMM. These charges are consistent with those obtained from B3LYP/6-31G (d,p) calculations snapshots from the MD simulations with CO_2_ adsorbed to a small water cluster (H_2_O)_10_ which yield *q*_C_ = 0.73*e* and *q*_O_ = − 0.35*e*. This compares with charges of *q*_C_ = 0.22*e* and *q*_O_ = − 0.21*e* for the CO molecule and *q*_O_ = − 0.1*e* for an oxygen atom adsorbed to (H_2_O)_10_. To assess the dependence of the results on the partial charges used, additional reactive MD simulations using the MMH parametrization were carried out with *q*_O_ = − 0.1*e* and *q*_C_ = 0.2*e* (i.e., *q*_CO_ = 0.1*e*) and with *q*_O_ = − 0.2*e* and *q*_C_ = 0.4*e* (i.e., *q*_CO_ = 0.2*e*). In all cases, recombination was found to speed up compared with *q*_CO_ = 0.3*e* and *q*_O_ = − 0.3*e* due to the increased mobility of the CO molecule and the O atom on the ASW when reduced partial charges are used.

The main focus of the present work is to study energy redistribution within the system following recombination of CO_A_ + O_B_ to form CO_2_. For this, the average total, translational and internal energy of the water molecules is analyzed for recombination on top of and inside ASW. Both, the time scale and amount of energy dissipated into translational and internal degrees of freedom was determined. The translational energy for the water molecules at each timestep was determined by first calculating the magnitude of the linear momentum of each water molecule from the stored velocities. From this the translational energy contribution is calculated for each water molecule considered and the total translational energy is accumulated. The internal energy for each water molecule is determined from the difference of the total kinetic energy and the translational energy.

## 3 Results and Discussion

In the following, the energy distribution in the water matrix of the ASW is separately discussed on the ∼100 ps and on the nanosecond time scale. Next, the energy flow away from the recombination site is analyzed and, finally, the energy redistribution to neighboring water molecules surrounding the recombination site is considered.

### 3.1 Recombination on the 100 ps Time Scale

**FIGURE 2:**
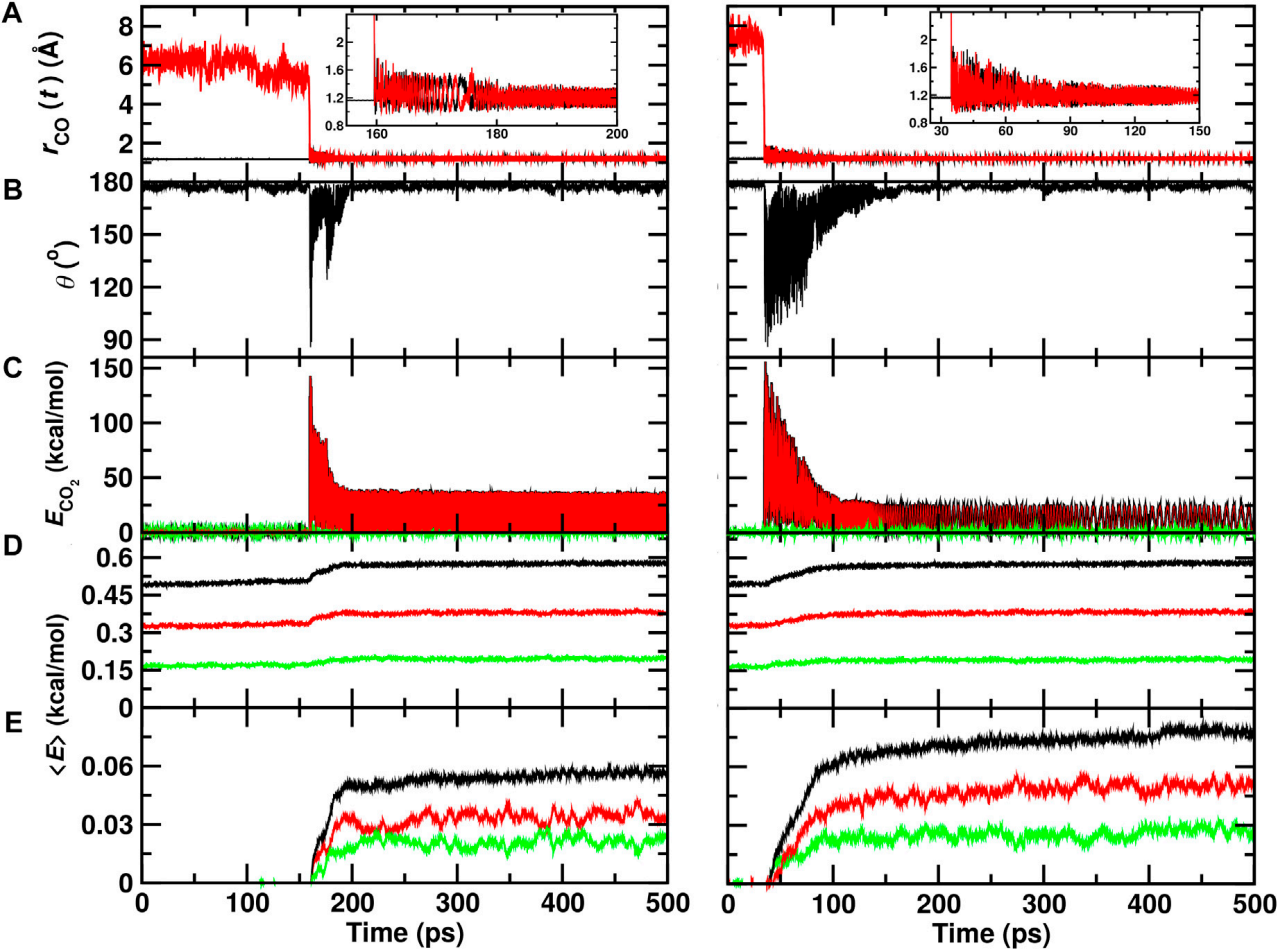
Recombination of O (^1^D)+CO(^1^Σ^+^) to form ground state CO2(Σg1) in the ASW cavity (left column) and on top of the ASW surface (right column). Initially, *R* = 6.0 Å and *θ* = 180°. **(A)**: O_B_–C_CO_ separation (red) and CO_A_ separation (black); **(B)**: the O-C-O angle *θ*; **(C–E)**: the average total (black), internal (red), and translational (green) energies for the CO_2_ molecule **(C)**, the average per water molecule **(D)**, and the magnitude of the average per water molecule relative to the energy before recombination **(E)**.

A typical trajectory for CO_A_ + O_B_ recombination inside the ASW cavity is shown in Figure 2 (left column). Initially, the C–O_B_ separation is ∼6 Å (Figure 2A). Within 150 ps recombination takes place and angular distortions lead to exploration of angles *θ*_OCO_ ∼ 90° (Figure 2B). Relaxation of the angle occurs within the following 50 ps and the CO_2_ molecule remains in an internally excited state on much longer time scales, see Figure 2C. (Upadhyay et al., 2021). Concomitantly, the average internal energy of the surrounding water molecules increases by about 10%, see black, red and green traces in Figures 2D,E. The translational (phononic) modes (green) acquire approximately 1/3 of the additional energy whereas the internal energy (red) increases by the remaining 2/3.

Figure 2 (right column) reports a recombination trajectory on top of ASW. In this case, recombination takes place after ∼35 ps and wide angular excursions extend out to 100 ps The amount of energy picked up by the water matrix is larger compared to recombination inside ASW (Figures 2D,E). The average total energy per water molecule increases by close to 20% and the amount that goes into internal degrees of freedom is considerably larger. For the translational modes, the energy after recombination is comparable to that for recombination within the cavity.

**FIGURE 3:**
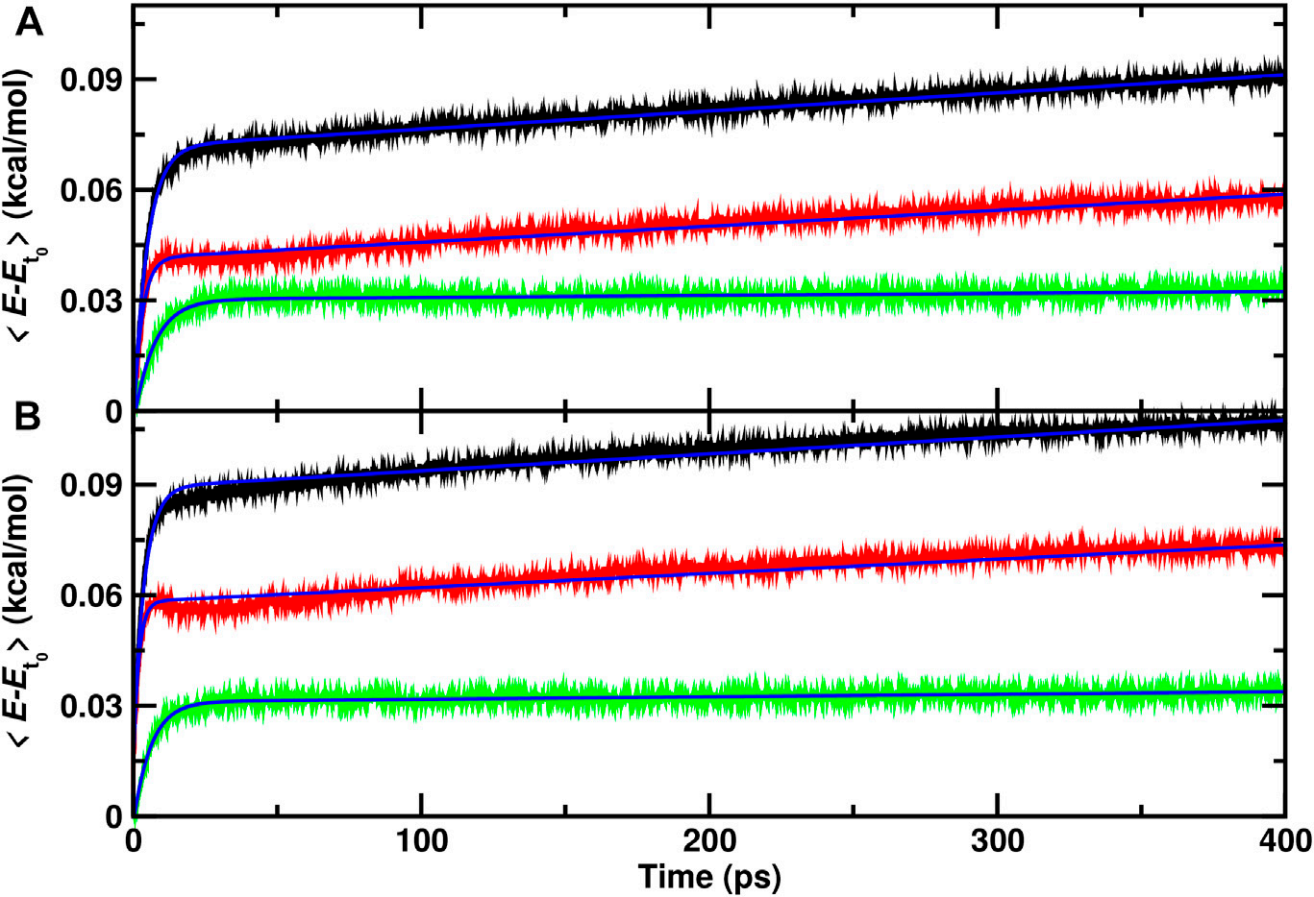
Average total (black), internal (red) and translational (green) energies for water over 70 independent runs relative to the average before recombination. The time of reaction for all trajectories is shifted to *t* =0 and defined by the first instance at which $r_{{\text{C}-\text{O}}_{\text{B}}}<1.6$ Å. **(A)**: recombination within the ASW cavity. **(B)**: recombination for CO_A_ + O_B_ on the top of the ASW surface. The blue solid line is a fit to an empirical expression $\epsilon =a_{0}e^{-t/a_{1}}+a_{2}t+a_{3}$ , see text.

From a set of 70 recombination trajectories for the reaction within the cavity and on top of the ASW surface, the averaged energy contents in translational, internal and all degrees of freedom of the water molecules were determined (Figure 3). For this analysis, the time of reaction was set to zero (*t* = 0) to align all reactive trajectories and all energies are reported relative to the averages before recombination. The translational contribution for recombination within and on top of ASW re-equilibrates on the ∼ 25 ps time scale after which no change in the phononic degrees of freedom is observed. Contrary to that, the internal degrees of freedom (red traces) show temporal evolution on two time scales: a rapid phase on the picosecond time scale, followed by a slow, long increase in the internal energies. This is also reflected in the averaged total energy (black).

As for the single trajectories, the amount of energy released from the recombination reaction into the translational degrees of freedom is similar for the reaction inside the cavity and on top of the ASW surface. For the internal degrees of freedom, however, recombination on top of the ASW surface leads on an average increase per water molecule by 0.075 kcal/mol within 400 ps (Figure 3B) compared with 0.06 kcal/mol for the process inside the cavity. Also, there is a characteristic decrease in the internal contribution for recombination on the surface after 15 ps which is even present when averaging over 70 independent runs. This feature is not found for recombination within ASW.

To estimate approximate time scales for the different processes involved, the average energies were fitted to an empirical expression $\epsilon =a_{0}e^{-t/a_{1}}+a_{2}t+a_{3}$ where *ϵ* is any of the energies considered. Such a functional form was chosen after inspection of the data in Figure 3 and accounts for the rapid initial increase in the three energies together with the slow variation of the internal energy on longer times. This parametrization is not able to model the dip around 15 ps for recombination on to of the surface, though. The time scales *a*_1_ for total, internal, and translational energies are (4.8, 2.9, 7.1) ps for recombination inside the cavity and speed up to (3.9, 1.9, 6.1) ps for the process on the ASW surface. It is of interest to note that the rapid time scale for the internal energy is considerably faster than the kinetics of the translational degrees of freedom for both types of recombinations. The parameter *a*_2_ which describes the slow increase of internal energy has a value of *a*_2_ = 4.3 × 10^–2^ (kcal/mol)/ns for recombination in the cavity and *a*_2_ = 3.8 × 10^–2^ (kcal/mol)/ns for the reaction on the surface, and is vanishingly small for the translational energy.

Average internal energies from representative independent runs for recombination inside the cavity and on top of the ASW surface are shown in Supplementary Figures S1, S2. For recombination inside the cavity (Supplementary Figure S1) the results confirm that the energy content in the internal degrees of freedom increases considerably faster than for the translation. Also, it is found that the amount of energy transferred to translation after recombination is smaller than that partitioned into internal degrees of freedom. For recombination on the ASW surface the same observations are made. In addition, the pronounced maximum after ∼5 ps is present in all examples shown in Supplementary Figure S2. To provide a molecularly resolved interpretation of this feature the HOH angle time series *θ*(*t*) was analyzed for a trajectory in which CO + O recombination occurred after 35 ps, see Supplementary Figure S3B. At the time of reaction the water bending angle decreases from its average equilibrium value by ⟨Δ*θ*⟩∼ 1° over the next 70 ps after which it relaxes back to the original value. The signature in the internal energy extends over ∼30 ps, see Supplementary Figure S2. Hence, it is possible that changes in the average water geometry following CO + O recombination are responsible for the overshooting and subsequent relaxation of the internal energy for the reaction on the surface. The HOH angle for a simulation within ASW in Supplementary Figure S3A also shows a slight adjustment of the valence angle after recombination. Contrary to the situation on the surface, the average angle does not relax to the value before recombination, though.

### 3.2 Recombination Dynamics on Longer Time Scales

**FIGURE 4:**
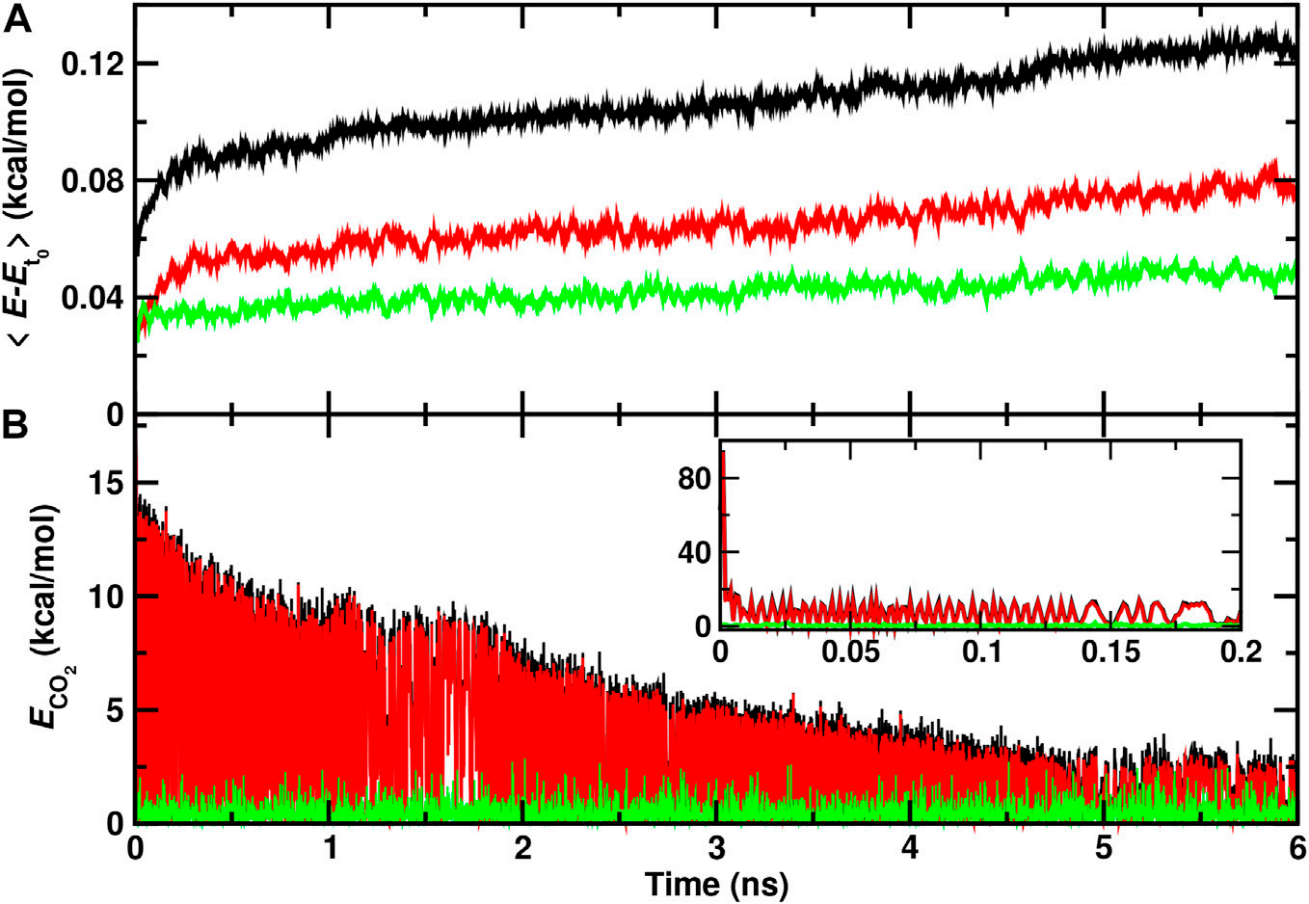
Total (black), internal (red), and translational (green) energies for water molecules **(A)** and for the recombined CO_2_ molecule **(B)** form a 6 ns rebinding trajectory on the top of the ASW surface. The time of reaction is shifted to *t* = 0 and defined by the first instance at which $r_{{\text{C}-\text{O}}_{\text{B}}}<1.6$ Å. CO_2_ continues to relax and the energy in the ASW further increases beyond the maximum simulation time of 6 ns after CO_2_ recombination.

It is also of interest to analyze the energy redistribution on the multi-nanosecond time scale. Figure 4A demonstrates that the average total kinetic energy per water molecule continuously increases even on the nanosecond time scale. Most of this increase is due to the internal degrees of freedom although the translational component also shows a continuous slow increase on the nanosecond time scale.

The relaxation of the CO_2_ internal energy is reported in Figure 4B. Within the first few picoseconds (inset) the internal energy is quenched to ∼10 kcal/mol after which two relaxations are observed. A first phase during 1 nanosecond following recombination and a second, slower phase extending out to 6 ns and beyond. By the end of the simulation the average internal energy of the CO_2_ molecule has decreased to ∼2.5 kcal/mol on average. Hence, it is expected that energy transfer to the surrounding water continues but slows down considerably on the 10 ns time scale and longer.

### 3.3 Energy Migration Around the Recombination Site

**FIGURE 5:**
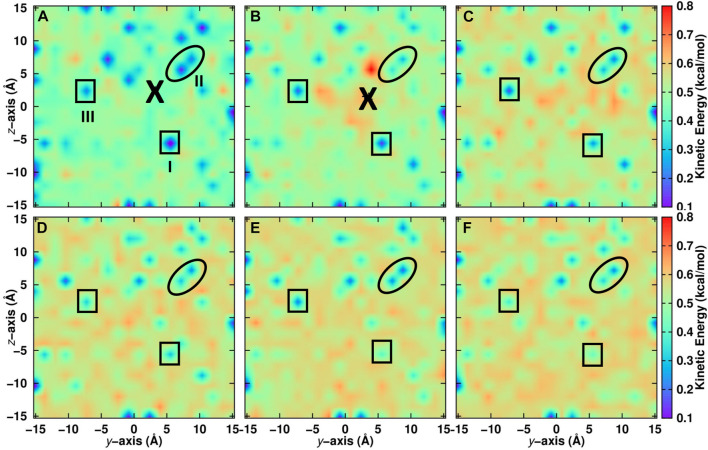
Kinetic energy of water molecules projected onto the (*y*, *z*) − plane averaged over seven independent simulations on the top layer of the ASW surface. Recombination of CO and O takes place at the location labelled with “X”. Before recombination **(A)**: − 5 ≤ *t* ≤ 0 ps) and after recombination **(B)**: 0 ≤ *t* ≤ 5 ps, **(C)**: 5 ≤ *t* ≤ 10 ps, **(D)**: 10 ≤ *t* ≤ 50 ps, **(E)**: 50 ≤ *t* ≤ 100 ps and **(F)**: 100 ≤ *t* ≤ 200 ps). The average position of CO is indicated by a large black cross. Noteworthy regions are labelled I to III and surrounded by solid lines. Region I is cool at early times and gradually warms up. Region II remains cool for most of the simulation time and region III alternates between cool and warm. For results within 10 Å of the surface, see Supplementary Figure S4.

For a positionally resolved picture of energy flow the simulation system was separated in voxels with dimension 31 × 1 × 1 Å^3^. The kinetic energy of all water molecules within one such voxel was averaged along the trajectory and projected onto the (*y*, *z*) − plane. Which water molecules belong to a particular voxel was decided based on the water-oxygen atom coordinates. Figure 5A reports the distribution of total kinetic energy distribution before recombination. The recombination site is at (*y* = 2, *z* = 2) Å and marked as a large cross. Within the first 5 ps after recombination the kinetic energy of water molecules within ∼ 10 Å of the recombination site increases considerably, by up to a factor of 4. Following this, energy redistributes continuously across the entire surface on the 200 ps time scale, see panels C to F.

Certain regions that are initially “cold” (blue)—e.g., the region labelled “I” at (*y* = 5, *z* = − 5) Å in Figure 5—warm up as energy transfer from CO_2_ to the water molecules occurs. Conversely, other regions remain “cool”, such as region “II” around (*y* = 5, *z* = 5) Å for which the color code remains blue until 200 ps. Yet for other regions, such as “III”, the total kinetic energy oscillates between cooler and warmer. It is also instructive to include only the first few ASW layers in this analysis which was done in Supplementary Figure S4. Here, the voxels have sizes 1 × 1 × 1 A^3^. For one, the cool regions are more extended before recombination. After recombination energy transfer occurs in a similar fashion as for the full system. However, the warm regions are less extended. This suggests that energy transfer also occurs to a considerable extent *into* the bulk rather than across the surface of the ASW even for recombination on top of ASW.

### 3.4 Energy Flow to Nearby Water Molecules

**FIGURE 6:**
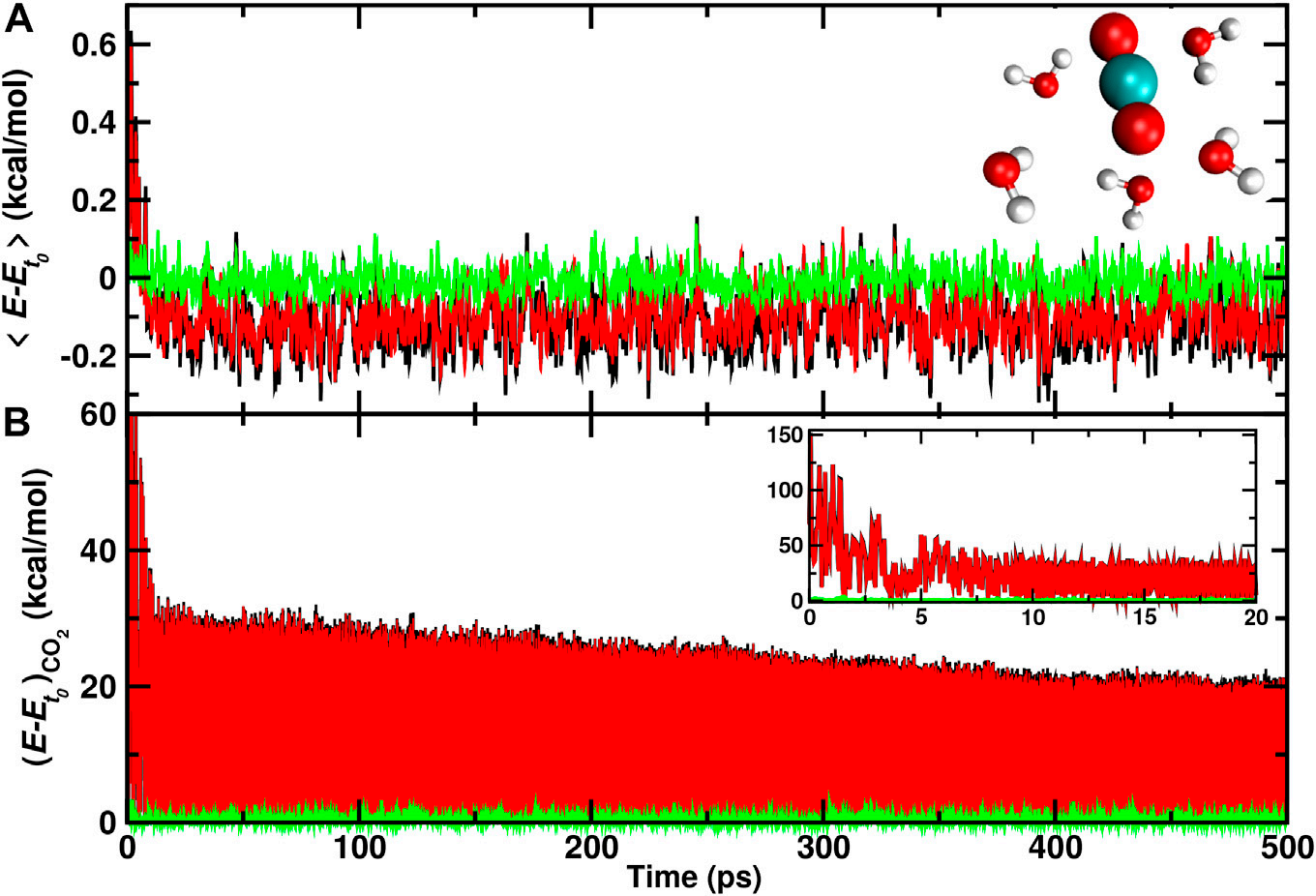
Average total (black), internal (red), and translational (green) energies for 5 water molecules **(A)** closest to the CO_2_, and the CO_2_ molecule **(B)** formed from a recombination trajectory on the ASW surface. The time of reaction is shifted to *t* = 0 and defined by the first instance at which $r_{{\text{C}-\text{O}}_{\text{B}}}<1.6$ Å. The initial time scale for energy redistribution from the relaxing CO_2_ molecule to internal and translational degrees of freedom of the ASW occurs on the 10 ps time scale with slow gradual relaxation on the ∼100 ps to ns time scale. On the 500 ps time scale the 5 water molecules slightly cool compared with the kinetic energy before recombination.

Finally, individual water molecules in immediate proximity of the recombination site are analyzed. For one trajectory with recombination on the ASW surface the average total, internal, and translational energies for the 5 water molecules closest to the recombination site are reported in Figure 6. During the first 10 ps after recombination the average total kinetic energy increases by up to 0.6 kcal/mol per water molecule. Conversely, the translational energy contribution fluctuates around zero which indicates that the local structure of ASW remains intact and most of the energy flows into internal degrees of freedom.

After this initial increase, cooling of these few nearby water molecules takes place with a long-time average of −0.1 kcal/mol per water molecule in the internal degrees of freedom. On the 500 ps no noticeable change in the translational energy content is observed. For the CO_2_ molecule (Figure 6B) the translational energy remains small throughout the trajectory whereas the internal energy decreases rapidly within the first 5 ps following recombination. Subsequently, slow gradual cooling on the 100 ps to nanosecond time scale takes place as was already found earlier, Figure 4.

## 4 Discussion and Conclusion

The present work reports on the energy redistribution across ASW following O(^1^D)+CO(^1^Σ^+^) recombination to form CO2(Σg+1) on the surface and in a cavity. It is found that energy distribution occurs in two phases, one on the picosecond and one on the nanosecond time scale for both locations. Although the time dependence of the processes is similar for the two different recombination sites (inside vs. on top), the dynamics differs in a number of ways. Firstly, recombination on the surface leads to excess internal energy on the picosecond time scale which subsequently relaxes and additional energy transfer into water modes occurs on longer time scales. Secondly, recombination within the cavity considered here leads to smaller magnitude (∼ 15*%*) of energy transferred per water molecule compared with the process on the surface (∼ 25*%*). A possible reason for this is that within a sufficiently large cavity the recombined CO_2_ molecule exchanges energy with the surrounding through direct collision whereas on the surface CO_2_ is always in contact with the ASW. In other words, the coupling between CO_2_ and water differs for recombination within ASW and on top of it. Finally, heating of the water molecules occurs on the 10 ps time scale following the recombination reaction. Consistent with earlier work, (Fredon et al., 2021; Upadhyay et al., 2021), no CO_2_ desorption is found from the simulations carried out here.

It is of interest to note that - ultimately - energy redistribution in such systems follows quantum mechanical principles. The present results suggest that the local energy generated from CO + O recombination is probably sufficient to excite internal modes of individual water molecules surrounding the recombination site. Hence, after CO + O recombination the ASW will be in a state characterized by a few internally and vibrationally excited water molecules embedded into a matrix of water molecules in the ground state. Earlier work on a related problem—the vibrational relaxation of a quantum oscillator coupled to oscillators of a biomolecule (Stock, 2009)—found that using classical mechanics leads to qualitatively correct results compared with a full quantum treatment. For the relaxation times a moderate factor of two for the difference between classical and rigorous quantum simulations was reported. Hence, for the present problem it is also expected that similar conclusions apply and that the nonequilibrium relaxation dynamics of individual vibrationally excited water molecules surrounded by vibrationally cold water molecules can be captured qualitatively from using classical dynamics.

In summary, the present work demonstrates that O(^1^D)+CO(^1^Σ^+^) recombination to form CO2(Σg+1) leads to excitation of both, phononic and internal modes of the water molecules that constitute the ASW. The time scales for this are on the pico- and nano-second and lead to warming the water matrix. Water molecules in direct proximity of the recombination site may become vibrationally excited and the time scale for their relaxation back to the ground state will depend on the coupling to the immediate environment. Full relaxation of the CO_2_ molecule is expected to require several 10–100 nanoseconds.

## Data Availability

The raw data supporting the conclusion of this article will be made available by the authors, without undue reservation. Data on the simulations and the PESs is available at: https://github.com/MMunibas/co2.asw and https://github.com/MMunibas/CO2-PESs.
