# Supplementary material for: Energy Redistribution Following CO2 Formation on Cold Amorphous Solid Water
Source: Front Chem. 2022 Feb 8;9:827085. doi: 10.3389/fchem.2021.827085 (PMC8861491; doi:10.3389/fchem.2021.827085)
Supplement: Supplementary file 1 [file DataSheet1.pdf]

Supporting Information: Energy Redistribution following CO_2 Formation on Cold Amorphous Solid Water

Meenu Upadhyay and Markus Meuwly*

Department of Chemistry, University of Basel, Klingelbergstrasse 80 , CH-4056 Basel, Switzerland.

E-mail: m.meuwly@unibas.ch

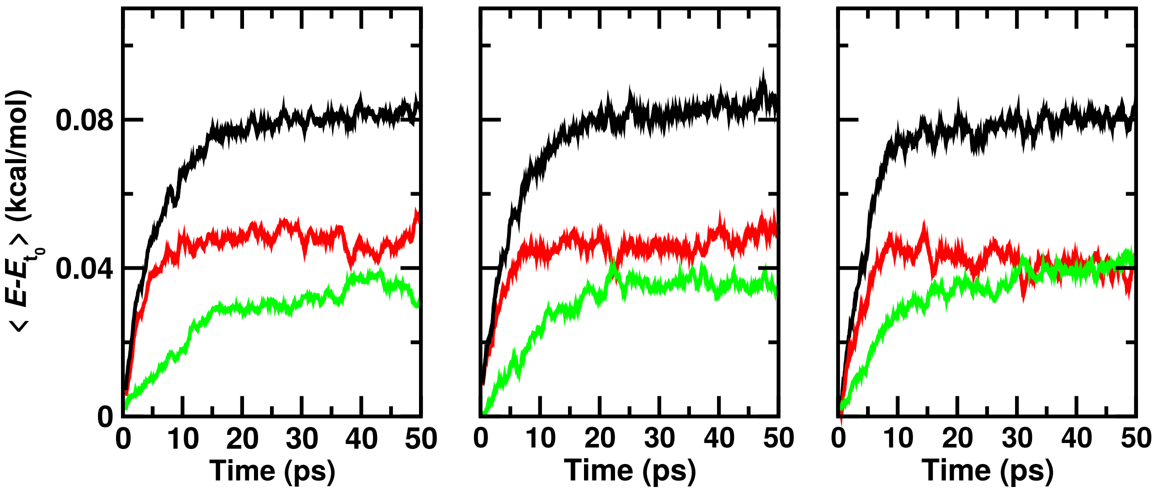

Figure S1: Total (black), internal (red), and translational (green) energies for water molecules from 3 independent simulations inside the ASW cavity. The time of reaction for all is shifted to $t = 0$ and defined by the first instance at which $r_{\mathrm{C-O_B}} < 1.6$ Å.

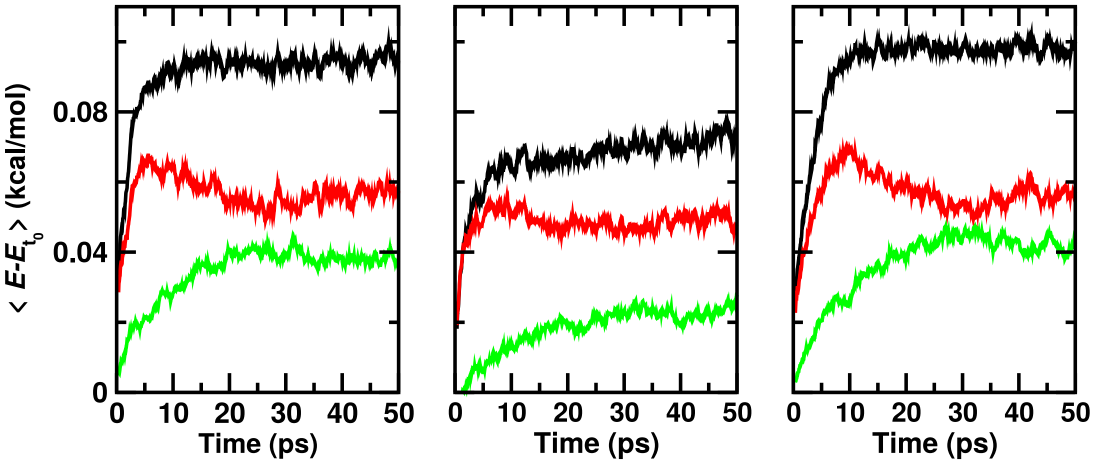

Figure S2: Total (black), internal (red), and translational (green) energies for water molecules from 3 independent simulations on the top of the ASW surface. The time of reaction for all is shifted to $t = 0$ and defined by the first instance at which $r_{\mathrm{C-O_B}} < 1.6$ Å.

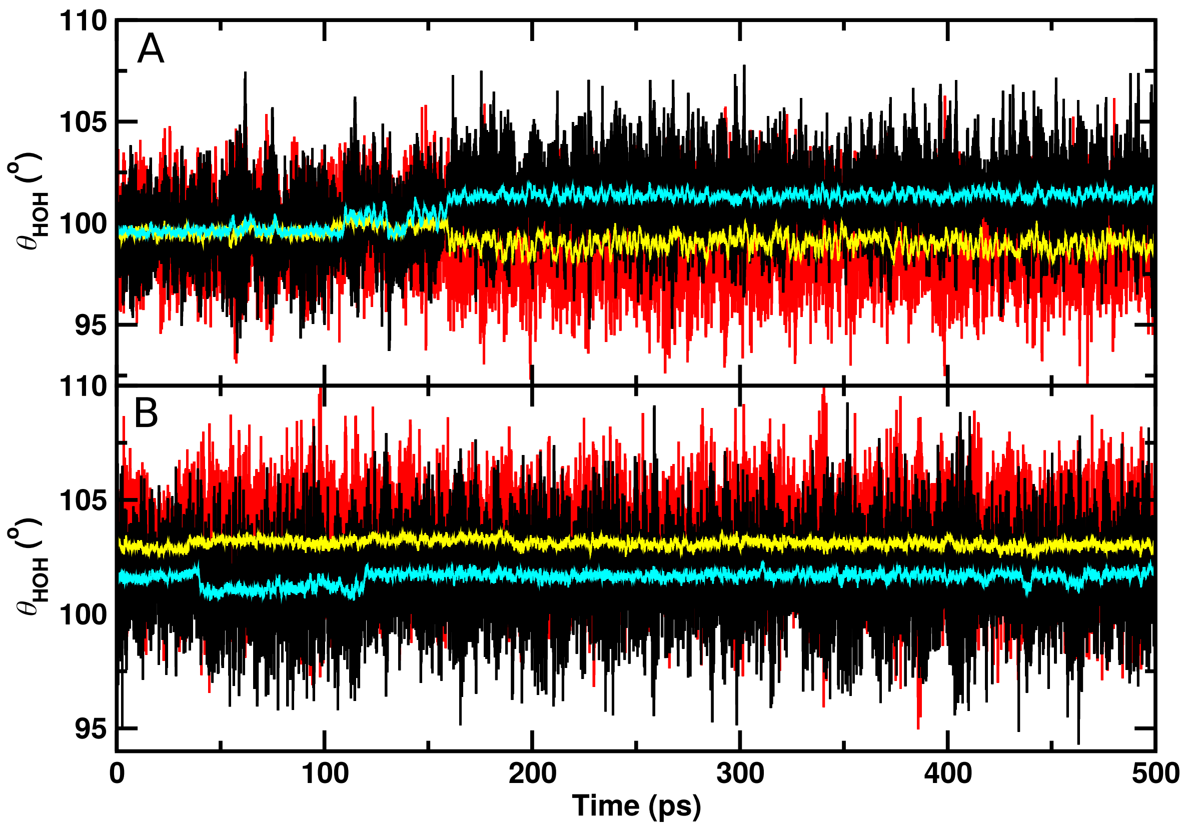

Figure S3: The bending angle time series for two water molecules closest to the recombined CO_2. Panel A: recombination inside the ASW cavity at 160 ps. Panel B: recombination on the water surface at 35 ps. Black/red lines are the raw data and cyan/yellow lines are the respective average over 20 neighboring points.

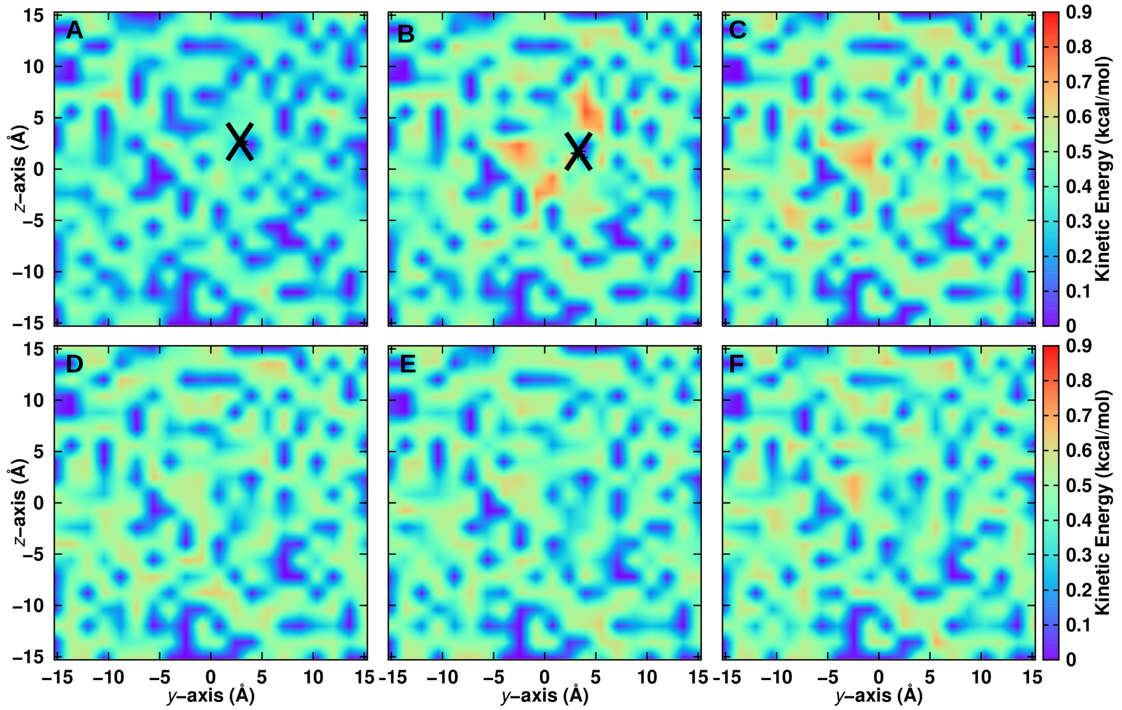

Figure S4: Kinetic energy of water molecules projected onto the $(y, z)-$plane averaged over 7 independent simulations on the top layer of ASW surface. The kinetic energy is averaged over voxels $10 \times 1 \times 1$ Å^3, i.e. only within 10 Å of the surface. Here, $t = 0$ is defined as the first instance at which $r_{\mathrm{C-O_B}} < 1.6$ Å and all times are aligned with respect to this reference. Before recombination (Panel A: $-5 \leq t \leq 0$ ps) and after recombination (Panel B: $0 \leq t \leq 5$ ps, Panel C: $5 \leq t \leq 10$ ps, Panel D: $10 \leq t \leq 50$ ps, Panel E: $50 \leq t \leq 100$ ps and Panel F: $100 \leq t \leq 200$ ps). The average position of CO is indicated by black filled circles.
